# Multiple-Degree-of-Freedom Modeling and Simulation for Seismic-Grade Sigma–Delta MEMS Capacitive Accelerometers

**DOI:** 10.3390/s23125394

**Published:** 2023-06-07

**Authors:** Xuefeng Wang, Penghao Zhang, Shijin Ding

**Affiliations:** State Key Laboratory of ASIC and System, School of Microelectronics, Fudan University, Shanghai 200433, China

**Keywords:** MEMS accelerometers, sigma–delta, multiple-degree-of-freedom, electromechanical modeling, noise

## Abstract

The high-order mechanical resonances of the sensing element in a high-vacuum environment can significantly degrade the noise and distortion performance of seismic-grade sigma–delta MEMS capacitive accelerometers. However, the current modeling approach is unable to evaluate the effects of high-order mechanical resonances. This study proposes a novel multiple-degree-of-freedom (MDOF) model to evaluate the noise and distortion induced by high-order mechanical resonances. Firstly, the MDOF dynamic equations of the sensing element are derived using the principle of modal superposition and Lagrange’s equations. Secondly, a fifth-order electromechanical sigma–delta system of the MEMS accelerometer is established in Simulink based on the dynamic equations of the sensing element. Then, the mechanism through which the high-order mechanical resonances degrade the noise and distortion performances is discovered by analyzing the simulated result. Finally, a noise and distortion suppression method is proposed based on the appropriate improvement in high-order natural frequency. The results show that the low-frequency noise drastically decreases from about −120.5 dB to −175.3 dB after the high-order natural frequency increases from about 130 kHz to 455 kHz. The harmonic distortion also reduces significantly.

## 1. Introduction

High-end capacitive accelerometers based on microelectromechanical system (MEMS) technology are widely applied in seismometers [1,2], inclination measurement [3], microgravity measurement [4], inertial navigation [5], etc. A high-resolution MEMS accelerometer with a noise floor of sub-µg$/\sqrt{Hz}$ is commonly required for seismic-grade application [1]. The total noise mainly consists of Brownian noise, quantization noise, and circuit noise [6].

Brownian noise can be lowered by improving the weight of the proof mass and the quality factor (Q) [7,8]. Thus, the sensing element of seismic-grade MEMS accelerometers is usually packaged in a vacuum to ensure high Q [8]. However, high Q causes the sensing element to exhibit unstable behavior, such as a long settling time and a significant overshoot. Hence, a closed-loop control system is necessary for the high Q sensing element to ensure stability [6]. The closed-loop solution based on the principle of electromechanical sigma–delta modulators (EM-ΣΔM) has been widely used in MEMS capacitive accelerometers, which can provide high-resolution digital output and possess advantages such as high linearity and wide bandwidth [9].

Henrion et al. first proposed a second-order EM-ΣΔM MEMS accelerometer in 1990, in which the sensing element was used as two cascaded integrators [10]. Kulah et al. presented a detailed noise study of second-order EM-ΣΔM MEMS accelerometers to achieve sub-μg resolution [11]. Second-order EM-ΣΔM accelerometers have relatively poor quantization noise shaping because the equivalent DC gain of the mechanical integrator is somewhat low [12]. Higher-order closed-loop sigma–delta accelerometers are proposed to improve the ability to perform quantization noise shaping. Amini et al. designed and implemented two cascaded switched-capacitor integrators with a sensing element to form a fourth-order EM-ΣΔM accelerometer, improving the system’s dynamic range by 20 dB [13]. Xu et al. implemented a fifth-order EM-ΣΔM accelerometer with a fully differential switched-capacitor interface to achieve 200 ng/√Hz input noise density [14]. Chen et al. proposed a sixth-order EM-ΣΔM accelerometer with a vacuum packaging sensing element based on distributed feedback loops [15].

The circuit noise is proportional to the input-referred noise of the interface circuit and inversely proportional to the capacitive sensitivity of the accelerometer [16]. A large proof-mass [16], a low spring constant [17], and high aspect ratio capacitive gaps [13] are usually adopted to improve the capacitive sensitivity. Additionally, it is necessary to optimize the interface circuit carefully, for example, by using the technique of correlated double sampling [18] and the feedforward noise reduction technique [19].

Besides the noises mentioned above, a new kind of noise has been reported in recent years. This noise originates from the interaction of the output bitstreams and MEMS high-order mechanical resonances in the electrostatic feedback force [20,21]. To evaluate this noise, the electromechanical model of the sensing element must include high-order natural modes. However, the conventional single-degree-of-freedom electromechanical (SDOF-EM) model employed in most studies only considers the fundamental natural mode [22]. Zhao et al. first used finite difference approximation to establish a multiple-degree-of-freedom model [22,23]. However, this model neglected the interaction of the output bitstreams and high-order mechanical resonances in the electrostatic feedback force. Thus, this model is still unable to evaluate the new noise.

This study proposes a multiple-degree-of-freedom electromechanical (MDOF-EM) model to evaluate the new noise resulting from high-order mechanical resonances. In Section 2, a MDOF-EM model is established based on the modal superposition and Lagrange’s equations. Then, the MDOF-EM model is combined with a lead compensator and a third-order modulator to form a systemic model for a fifth-order EM-ΣΔM MEMS accelerometer. Finally, the systemic model is simulated to analyze the effect of high-order mechanical resonances on noise and distortion performance and identify its method of suppression.

## 2. MDOF-EM Model of the Sensing Element

This section establishes the MDOF-EM model of the sensing element using the principle of modal superposition and Lagrange’s equations. Firstly, the description of finger displacement is studied.

### 2.1. Description of Finger Displacement

In most studies [5,6,7,8,9,12,13,15,16,17,24,25,26,27,28], finger flexibility is ignored to assume the sensing element of the seismic-grade MEMS accelerometer as a single-degree-of-freedom (SDOF) mass–damper–spring system. However, this conventional approach is unable to capture the effects of high-order mechanical resonances. After the sensing element has been packaged in a high-vacuum environment, the high-order natural modes are easily excited into resonance. The bending of fingers must also be considered to capture the effects of high-order mechanical resonances. Thus, it is crucial to describe the finger displacement.

**Figure 1:**
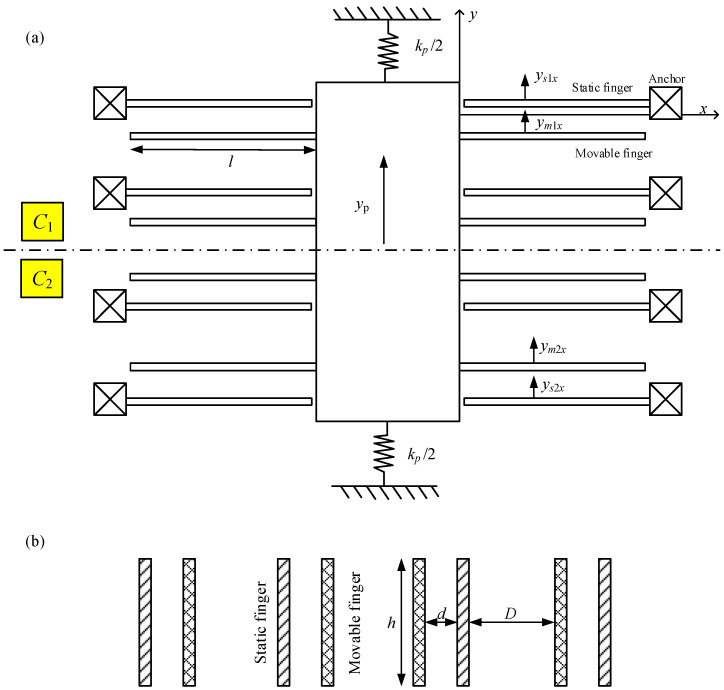
Structural diagram of the MEMS accelerometer. (**a**) Top view. (**b**) Cross-section view of fingers.

This study establishes the coordinate system shown in Figure 1 for the fingers. The origin of the coordinates is at the clamped end of the movable fingers, while the slight overlapping shift between movable and static fingers is neglected. The static fingers are typical cantilevers tied to the anchor, whereas the movable fingers are cantilevers moving with the rigid proof mass. Thus, according to the principle of modal superposition [29], the displacements of the static and movable fingers are expressed as

(1) $$y_{s1x}=G_{s}^{T}y_{s1},y_{s2x}=G_{s}^{T}y_{s2},y_{m1x}=y_{p}+G_{m}^{T}y_{m1},y_{m2x}=y_{p}+G_{m}^{T}y_{m2}$$

where *y_s_*_1*x*_ and *y_s_*_2*x*_ denote the displacements of the static fingers belonging to capacitors *C*_1_ and *C*_2_, respectively; *y_m_*_1*x*_ and *y_m_*_2*x*_ denote the displacements of the movable fingers belonging to capacitors *C*_1_ and *C*_2_, respectively; and *y_p_* indicates the displacement of the rigid proof mass. *G_s_* and *G_m_* represent the column vectors of mode shape for movable and static fingers, respectively, and depend only on the coordinate *x*. The column vectors of mechanical modal amplitudes *y_s_*_1_, *y_s_*_2_, *y_m_*_1_, and *y_m_*_2_ rely only on time. In this study, the mode shapes that equal one at the free end are adopted.

### 2.2. Electromechanical Dynamic Equations

The dynamic equations of the sensing element are derived from Lagrange’s equations. According to the principle of Lagrange’s equations for general electromechanical systems [30], Lagrange’s equations for an electrostatic actuator are expressed as

(2) $$\begin{array}{l} \frac{d}{dt}\left(\frac{\partial L}{\partial {\dot{q}}_{i}}\right)+\frac{\partial D_{c}}{\partial {\dot{q}}_{i}}-\frac{\partial L}{\partial q_{i}}=Q_{i}\\ L=T+W_{e}-V \end{array}$$

where *L* denotes the Lagrangian of the system, *D_c_* denotes the dissipation function, *Q_i_* denotes the generalized force, *q_i_* denotes the generalized coordinate, and *T*, *V*, and *W_e_* represent the kinetic, potential, and electrical energy, respectively.

#### 2.2.1. Energy Function

The kinetic energy of the system is expressed as

(3) $$T=\frac{1}{2}m_{p}{\dot{y}}_{p}^{2}+N\left(\int_{0}^{l}\frac{1}{2}\rho_{m}{\dot{y}}_{m1x}^{2}dx+\int_{0}^{l}\frac{1}{2}\rho_{m}{\dot{y}}_{m2x}^{2}dx+\int_{0}^{l}\frac{1}{2}\rho_{s}{\dot{y}}_{s1x}^{2}dx+\int_{0}^{l}\frac{1}{2}\rho_{s}{\dot{y}}_{s2x}^{2}dx\right)$$

where *m_p_* denotes the mass of the proof-mass, *N* denotes the number of movable or static fingers in capacitor *C*_1_ or *C*_2_, and *ρ_m_* and *ρ_s_* represent the mass per unit length of the movable and static fingers, respectively. Using Equations (1) and (3) leads to

(4) $$T=\frac{1}{2}m_{t}{\dot{y}}_{p}^{2}+{\dot{y}}_{p}\lambda_{m}^{T}\left({\dot{y}}_{m1}+{\dot{y}}_{m2}\right)+\frac{1}{2}{\dot{y}}_{m1}^{T}\gamma_{m}{\dot{y}}_{m1}+\frac{1}{2}{\dot{y}}_{m2}^{T}\gamma_{m}{\dot{y}}_{m2}+\frac{1}{2}{\dot{y}}_{s1}^{T}\gamma_{s}{\dot{y}}_{s1}+\frac{1}{2}{\dot{y}}_{s2}^{T}\gamma_{s}{\dot{y}}_{s2}$$

where *m_t_* denotes the mass sum of the proof mass and all movable fingers, and the equivalent mass *λ_m_*, *γ_m_*, and *γ_s_* are expressed as

(5) $$\lambda_{m}=N\int_{0}^{l}\rho_{m}G_{m}dx,\gamma_{m}=N\int_{0}^{l}\rho_{m}G_{m}G_{m}^{T}dx,\gamma_{s}=N\int_{0}^{l}\rho_{s}G_{s}G_{s}^{T}dx$$

In this study, the fingers’ bending is described by the Euler–Bernoulli beam theory, which assumes that the cross-section remains orthogonal to the neutral axis after bending [31]. Thus, the potential energy of the system is expressed as

(6) $$V=\frac{1}{2}k_{p}y_{p}^{2}+\frac{N}{2}\int_{0}^{l}EI_{m}{\left({y^{\prime \prime }}_{m1x}\right)}^{2}dx+\frac{N}{2}\int_{0}^{l}EI_{m}{\left({y^{\prime \prime }}_{m2x}\right)}^{2}dx+\frac{N}{2}\int_{0}^{l}EI_{s}{\left({y^{\prime \prime }}_{s1x}\right)}^{2}dx+\frac{N}{2}\int_{0}^{l}EI_{s}{\left({y^{\prime \prime }}_{s2x}\right)}^{2}dx$$

where *k_p_* denotes the stiffness of the spring supporting the proof-mass; *E* denotes Young’s modulus; *I_m_* and *I_s_* denote the moment of inertia of the cross-section in movable and static fingers, respectively; and ″ represents the second derivative to the coordinate *x*. Using Equation (1) with (6), the potential energy of the system is expressed as

(7) $$V=\frac{1}{2}k_{p}y_{p}^{2}+\frac{1}{2}y_{m1}^{T}k_{m}y_{m1}+\frac{1}{2}y_{m2}^{T}k_{m}y_{m2}+\frac{1}{2}y_{s1}^{T}k_{s}y_{s1}+\frac{1}{2}y_{s2}^{T}k_{s}y_{s2}$$

where the equivalent stiffness values *k_m_* and *k_s_* for movable and static fingers are expressed as

(8) $$k_{m}=N\int_{0}^{l}EI_{m}{G^{\prime \prime }}_{m}{G^{\prime \prime }}_{m}^{T}dx,k_{s}=N\int_{0}^{l}EI_{s}{G^{\prime \prime }}_{s}{G^{\prime \prime }}_{s}^{T}dx$$

when the fingers are bent, the capacitances can be computed via integration after neglecting the fringe effect.

(9) $$\begin{array}{l} C_{1}=N\left[\int_{0}^{l}\frac{\varepsilon h}{d-y_{p}-G_{m}^{T}y_{m1}+G_{s}^{T}y_{s1}}dx+\int_{0}^{l}\frac{\varepsilon h}{D+y_{p}+G_{m}^{T}y_{m1}-G_{s}^{T}y_{s1}}dx\right]\\ C_{2}=N\left[\int_{0}^{l}\frac{\varepsilon h}{d+y_{p}+G_{m}^{T}y_{m2}-G_{s}^{T}y_{s2}}dx+\int_{0}^{l}\frac{\varepsilon h}{D-y_{p}-G_{m}^{T}y_{m2}+G_{s}^{T}y_{s2}}dx\right] \end{array}$$

where *d* and *D* denote the gaps between the movable and static fingers, *h* denotes the height of the fingers, and *ε* represents the permittivity of the air, as shown in Figure 1. This study uses EM-ΣΔM to realize a digital output of the seismic-grade MEMS accelerometer. According to the feedback principle of the EM-ΣΔM accelerometer [32,33], the electrical energy is expressed as

(10) $$W_{e}=\frac{1}{2}C_{1}{\left(V_{f}S_{o}-V_{f}\right)}^{2}+\frac{1}{2}C_{2}{\left(V_{f}S_{o}+V_{f}\right)}^{2}$$

where *V_f_* denotes the feedback voltage, and *S_o_*∈(−1, 1) represents the one-bit output bitstream, which controls the applied direction of feedback voltage.

#### 2.2.2. Dissipative Function

The damping coefficient of MEMS accelerometers mainly results from the squeeze-film viscous damping between the movable fingers and the static ones. It can be evaluated based on the quality factor *Q* [34]. The squeeze-film damping is directly proportional to the total length of all movable fingers [35], so the damping coefficient per unit finger’s length can be expressed as

(11) $$c_{u}=\sqrt{m_{t}k_{p}}/\left(2NlQ\right)$$

According to the principle of the quadratic dissipative function [30], the dissipative function of the system is expressed as

(12) $$D_{c}=N\left(\int_{0}^{l}\frac{1}{2}c_{u}{\dot{y}}_{m1x}^{2}dx+\int_{0}^{l}\frac{1}{2}c_{u}{\dot{y}}_{s1x}^{2}dx\right)+N\left(\int_{0}^{l}\frac{1}{2}c_{u}{\dot{y}}_{m2x}^{2}dx+\int_{0}^{l}\frac{1}{2}c_{u}{\dot{y}}_{s2x}^{2}dx\right)$$

Using Equation (1) with (12) leads to

(13) $$D_{c}=\frac{1}{2}c{\dot{y}}_{p}^{2}+\left({\dot{y}}_{p}\eta_{m}^{T}{\dot{y}}_{m1}+\frac{1}{2}{\dot{y}}_{m1}^{T}\xi_{m}{\dot{y}}_{m1}+\frac{1}{2}{\dot{y}}_{s1}^{T}\xi_{s}{\dot{y}}_{s1}\right)+\left({\dot{y}}_{p}\eta_{m}^{T}{\dot{y}}_{m2}+\frac{1}{2}{\dot{y}}_{m2}^{T}\xi_{m}{\dot{y}}_{m2}+\frac{1}{2}{\dot{y}}_{s2}^{T}\xi_{s}{\dot{y}}_{s2}\right)$$

where *η_m_*, *ξ_m_,* and *ξ_s_* denote the equivalent damping coefficient and are expressed as

(14) $$\eta_{m}=N\int_{0}^{l}c_{u}G_{m}dx,\xi_{m}=N\int_{0}^{l}c_{u}G_{m}G_{m}^{T}dx,\xi_{s}=N\int_{0}^{l}c_{u}G_{s}G_{s}^{T}dx$$

#### 2.2.3. Virtual Work Contributed by the Inertial Force

The virtual work contributed by the inertial force includes that from proof-mass, movable fingers, and static fingers.

(15) $$\delta W_{i}=-m_{p}a\delta y_{p}+N\int_{0}^{l}-\rho_{m}a\delta y_{m1x}dx+N\int_{0}^{l}-\rho_{m}a\delta y_{m2x}dx+N\int_{0}^{l}-\rho_{s}a\delta y_{s1x}dx+N\int_{0}^{l}-\rho_{s}a\delta y_{s2x}dx$$

Using Equation (1) with (15) leads to

(16) $$\delta W_{i}=-\delta y_{p}\left(m_{t}a\right)-\delta y_{m1}^{T}\left(\lambda_{m}a\right)-\delta y_{m2}^{T}\left(\lambda_{m}a\right)-\delta y_{s1}^{T}\left(\lambda_{s}a\right)-\delta y_{s2}^{T}\left(\lambda_{s}a\right)$$

where the equivalent mass *λ*_s_ is expressed as

(17) $$\lambda_{s}=N\int_{0}^{l}\rho_{s}G_{s}dx$$

According to the principle of generalized forces [30], the generalized force vector from Equation (16) is expressed as

(18) $$Q={\left[\begin{array}{ccccc} -m_{t}a & -\lambda_{m}a & -\lambda_{m}a & -\lambda_{s}a & -\lambda_{s}a \end{array}\right]}^{T}$$

#### 2.2.4. Dynamic Equations

According to the dynamics of the electromechanical system, when voltage sources are directly applied to the capacitors without resistors and inductors, only the dynamic equations of the mechanical part are applicable [30]. The electrical equation represents the simple charging of capacitors, i.e., *q* = *CV*. The charging aims to provide electrostatic feedback force, which has been included in mechanical dynamic equations. In other words, the charging affects the system’s performance by changing the electrostatic feedback force. Thus, just as in the previously reported studies [13,32], the electrical equation of charging is not expressed in this study.

Using Equations (4), (7), (10), (13), and (18) with (2), the dynamic equations of the system are expressed as

(19) $$M\ddot{y}+D\dot{y}+Ky=Q+F_{e}$$

where the amplitude vector *y*, mass matrix *M*, damping matrix *D*, stiffness matrix *K*, and electrostatic force vector *F_e_* are expressed as

(20) $$y=\left[\begin{array}{c} y_{p}\\ y_{m1}\\ y_{m2}\\ y_{s1}\\ y_{s2} \end{array}\right],M=\left[\begin{array}{ccccc} m_{t} & \lambda_{m}^{T} & \lambda_{m}^{T} & & \\ \lambda_{m} & \gamma_{m} & & & \\ \lambda_{m} & & \gamma_{m} & & \\ & & & \gamma_{s} & \\ & & & & \gamma_{s} \end{array}\right],D=\left[\begin{array}{ccccc} c & \eta_{m}^{T} & \eta_{m}^{T} & & \\ \eta_{m} & \xi_{m} & & & \\ \eta_{m} & & \xi_{m} & & \\ & & & \xi_{s} & \\ & & & & \xi_{s} \end{array}\right],K=\left[\begin{array}{ccccc} k_{p} & & & & \\ & k_{m} & & & \\ & & k_{m} & & \\ & & & k_{s} & \\ & & & & k_{s} \end{array}\right],F_{e}=\left[\begin{array}{c} F_{ep}\\ F_{em1}\\ F_{em2}\\ F_{es1}\\ F_{es2} \end{array}\right]$$

The elements of the electrostatic force vector are expressed as

(21) $$\begin{array}{l} F_{ep}=F_{e0}{\left(S_{o}-1\right)}^{2}\left[1/{\left(1-{\tilde{y}}_{p}\right)}^{2}-1/{\left(\tilde{D}+{\tilde{y}}_{p}\right)}^{2}+2\left(r_{m}^{T}{\tilde{y}}_{m1}-r_{s}^{T}{\tilde{y}}_{s1}\right)/{\left(1-{\tilde{y}}_{p}\right)}^{3}\right]\\ -F_{e0}{\left(S_{o}+1\right)}^{2}\left[1/{\left(1+{\tilde{y}}_{p}\right)}^{2}-1/{\left(\tilde{D}-{\tilde{y}}_{p}\right)}^{2}-2\left(r_{m}^{T}{\tilde{y}}_{m2}-r_{s}^{T}{\tilde{y}}_{s2}\right)/{\left(1+{\tilde{y}}_{p}\right)}^{3}\right] \end{array}$$

(22) $$F_{em1}=F_{e0}{\left(S_{o}-1\right)}^{2}\left[r_{m}/{\left(1-{\tilde{y}}_{p}\right)}^{2}-r_{m}/{\left(\tilde{D}+{\tilde{y}}_{p}\right)}^{2}+2\left(r_{mm}{\tilde{y}}_{m1}-r_{ms}{\tilde{y}}_{s1}\right)/{\left(1-{\tilde{y}}_{p}\right)}^{3}\right]$$

(23) $$F_{em2}=-F_{e0}{\left(S_{o}+1\right)}^{2}\left[r_{m}/{\left(1+{\tilde{y}}_{p}\right)}^{2}-r_{m}/{\left(\tilde{D}-{\tilde{y}}_{p}\right)}^{2}-2\left(r_{mm}{\tilde{y}}_{m2}-r_{ms}{\tilde{y}}_{s2}\right)/{\left(1+{\tilde{y}}_{p}\right)}^{3}\right]$$

(24) $$F_{es1}=-F_{e0}{\left(S_{o}-1\right)}^{2}\left[r_{s}/{\left(1-{\tilde{y}}_{p}\right)}^{2}-r_{s}/{\left(\tilde{D}+{\tilde{y}}_{p}\right)}^{2}+2\left(r_{sm}{\tilde{y}}_{m1}-r_{ss}{\tilde{y}}_{s1}\right)/{\left(1-{\tilde{y}}_{p}\right)}^{3}\right]$$

(25) $$F_{es2}=F_{e0}{\left(S_{o}+1\right)}^{2}\left[r_{s}/{\left(1+{\tilde{y}}_{p}\right)}^{2}-r_{s}/{\left(\tilde{D}-{\tilde{y}}_{p}\right)}^{2}-2\left(r_{sm}{\tilde{y}}_{m2}-r_{ss}{\tilde{y}}_{s2}\right)/{\left(1+{\tilde{y}}_{p}\right)}^{3}\right]$$

where the remaining electrostatic force *F*_e0_, normalized wide gap $\tilde{D}$, normalized displacements ${\tilde{y}}_{p},\;{\tilde{y}}_{m1},\;{\tilde{y}}_{m2},\;{\tilde{y}}_{s1},\;{\tilde{y}}_{s2}$, and length coefficients *r_m_*, *r_s_*, *r_mm_*, *r_ms_*, *r_sm_*, and *r_ss_* are expressed as

(26) $$F_{e0}=\frac{N\varepsilon hV_{f}^{2}l}{2d^{2}}$$

(27) $$\tilde{D}=D/d,{\tilde{y}}_{p}=y_{p}/d,{\tilde{y}}_{m1}=y_{m1}/d,{\tilde{y}}_{m2}=y_{m2}/d,{\tilde{y}}_{s1}=y_{s1}/d,{\tilde{y}}_{s2}=y_{s2}/d$$

(28) $$r_{m}=\frac{1}{l}\int_{0}^{l}G_{m}dx,r_{s}=\frac{1}{l}\int_{0}^{l}G_{s}dx,r_{mm}=\frac{1}{l}\int_{0}^{l}G_{m}G_{m}^{T}dx,r_{ms}=\frac{1}{l}\int_{0}^{l}G_{m}G_{s}^{T}dx,r_{sm}=\frac{1}{l}\int_{0}^{l}G_{s}G_{m}^{T}dx,r_{ss}=\frac{1}{l}\int_{0}^{l}G_{s}G_{s}^{T}dx$$

In Equations (21)–(25), because the normalized finger displacements ${\tilde{y}}_{m1}\sim {\tilde{y}}_{s2}$ are much smaller than 1, the dependence of electrostatic forces on normalized finger displacements is approximated linearly to improve the simulation speed.

If ignoring the flexibility of the fingers, i.e., the normalized finger displacements ${\tilde{y}}_{m1}\sim {\tilde{y}}_{s2}$ are assumed to be zero, the MDOF-EM model given in Equation (19) degrades into the SDOF-EM model that is widely adopted in current studies [5,6,7,8,9,12,13,15,16,17,24,25,26,27,28].

(29) $$m_{t}\ddot{y}+c\dot{y}+k_{p}y=-m_{t}a+\frac{F_{e0}{\left(S_{o}-1\right)}^{2}}{{\left(1-{\tilde{y}}_{p}\right)}^{2}}-\frac{F_{e0}{\left(S_{o}-1\right)}^{2}}{{\left(\tilde{D}+{\tilde{y}}_{p}\right)}^{2}}-\frac{F_{e0}{\left(S_{o}+1\right)}^{2}}{{\left(1+{\tilde{y}}_{p}\right)}^{2}}+\frac{F_{e0}{\left(S_{o}+1\right)}^{2}}{{\left(\tilde{D}-{\tilde{y}}_{p}\right)}^{2}}$$

### 2.3. Differential Capacitance

Using linear approximation for Equation (9), the differential capacitance between *C*_1_ and *C*_2_ is approximately

(30) $$\Delta C=\frac{N\varepsilon hl}{d}\left[\frac{1}{1-{\tilde{y}}_{p}}-\frac{1}{1+{\tilde{y}}_{p}}+\frac{1}{\tilde{D}+{\tilde{y}}_{p}}-\frac{1}{\tilde{D}-{\tilde{y}}_{p}}+\frac{r_{m}^{T}{\tilde{y}}_{m1}-r_{s}^{T}{\tilde{y}}_{s1}}{{\left(1-{\tilde{y}}_{p}\right)}^{2}}-\frac{r_{m}^{T}{\tilde{y}}_{m1}-r_{s}^{T}{\tilde{y}}_{s1}}{{\left(\tilde{D}+{\tilde{y}}_{p}\right)}^{2}}+\frac{r_{m}^{T}{\tilde{y}}_{m2}-r_{s}^{T}{\tilde{y}}_{s2}}{{\left(1+{\tilde{y}}_{p}\right)}^{2}}-\frac{r_{m}^{T}{\tilde{y}}_{m2}-r_{s}^{T}{\tilde{y}}_{s2}}{{\left(\tilde{D}-{\tilde{y}}_{p}\right)}^{2}}\right]$$

If the normalized finger displacements ${\tilde{y}}_{m1}\sim {\tilde{y}}_{s2}$ are assumed to be zero, the differential capacitance for the SDOF-EM model is acquired:

(31) $$\Delta C=\frac{N\varepsilon hl}{d}\left[\frac{1}{1-{\tilde{y}}_{p}}-\frac{1}{1+{\tilde{y}}_{p}}+\frac{1}{\tilde{D}+{\tilde{y}}_{p}}-\frac{1}{\tilde{D}-{\tilde{y}}_{p}}\right]$$

### 2.4. Selection of Finger Mode Order

**Figure 2:**
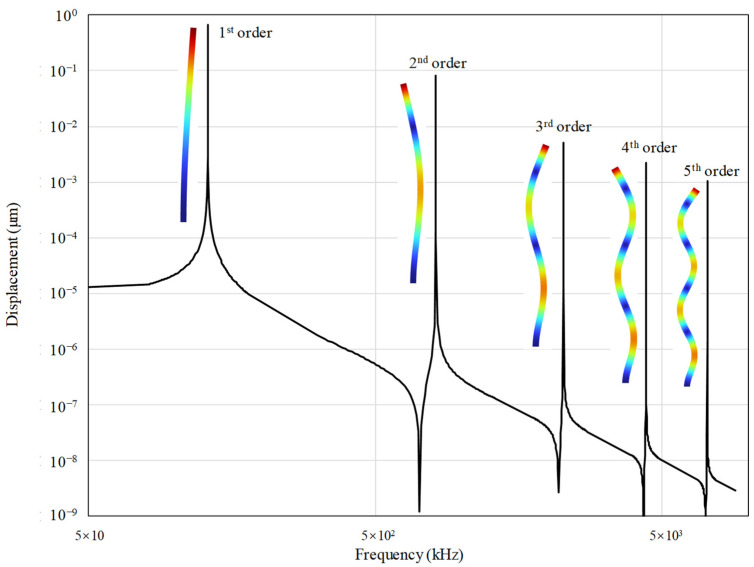
The frequency domain analysis results of a finger. The vertical axis represents the mean transverse displacement.

If more natural modes of the fingers are adopted, the precision of the MDOF-EM model will increase. However, more modes result in more degrees of freedom and a slower simulation speed of the EM-ΣΔM system. In this study, the frequency domain simulation of a finger is used to determine the mode number. The material properties and dimensions are listed in Table 1. The driving acceleration of the finger is 1 g, and the damping is 3.76 × 10^−6^ N*s/m^2^. The simulation result is shown Figure 2. The displacements generated by the second- to fifth-order modes are minimal compared with that of the first-order mode. In other words, the error remains small even though only the first-order mode is selected for the MDOF-EM model. However, the complexity of the model can decrease significantly. Therefore, this study only chooses the first-order mode of the fingers.

### 2.5. Parameters for Fingers with Uniform Rectangle Cross-Section

For the fingers with a uniform rectangle cross-section, which are widely used in current research, the normalized modal shape is expressed as [29]

(32) $$G_{m}=\frac{\cosh\left(1.875x/l\right)-\cos\left(1.875x/l\right)-0.734\left(\sinh\left(1.875x/l\right)-\sin\left(1.875x/l\right)\right)}{2}$$

(33) $$G_{s}=\frac{\cosh\left(1.875\left(l-x\right)/l\right)-\cos\left(1.875\left(l-x\right)/l\right)-0.734\left(\sinh\left(1.875\left(l-x\right)/l\right)+\sin\left(1.875\left(l-x\right)/l\right)\right)}{2}$$

Incorporating Equations (32) and (33) into (5), (8), (14), (17), and (28), the equivalent mass, stiffness, damping coefficient, and length coefficients are expressed as

(34) $$\lambda_{m}=\lambda_{s}=0.39N\rho whl,\gamma_{m}=\gamma_{s}=0.25N\rho whl$$

(35) $$k_{m}=k_{s}=0.254NEw^{3}h/l^{3}$$

(36) $$\eta_{m}=0.39Nc_{u}l,\xi_{m}=\xi_{s}=0.25Nc_{u}l$$

(37) $$r_{m}=r_{s}=0.39,r_{mm}=r_{ss}=0.25,r_{ms}=r_{sm}=0.061$$

where *w* denotes the width of the movable and static fingers, and *ρ* represents the density.

## 3. EM-ΣΔM System Based on MDOF-EM Model of the Sensing Element

This section establishes the EM-ΣΔM system of the seismic-grade MEMS accelerometer using an MDOF-EM model of the sensing element. In the EM-ΣΔM system, the MDOF-EM model is described using the state-space equation:

(38) $$\dot{z}=\left[\begin{array}{cc} 0 & I\\ -M^{-1}K & -M^{-1}D \end{array}\right]z+\left[\begin{array}{c} 0\\ M^{-1}\left(Q+F_{e}\right) \end{array}\right]$$

in which the state vector is expressed as

(39) $$z={\left[y_{p},y_{m1},y_{m2},y_{s1},y_{s2},{\dot{y}}_{p},{\dot{y}}_{m1},{\dot{y}}_{m2},{\dot{y}}_{s1},{\dot{y}}_{s2}\right]}^{T}$$

There is hope that the higher-order EM-ΣΔM system can be used to improve the shaping performance for quantization noise. However, the higher-order EM-ΣΔM system has disadvantages, such as a higher risk of instability and a more complex circuit [9]. The fifth-order EM-ΣΔM system has much better noise shaping performance than the second-, third-, and fourth-order systems, and can guarantee its stability [12]. However, an EM-ΣΔM system with an order higher than five does not further improve the noise performance [9]. Thus, a fifth-order EM-ΣΔM system is established to study the effect of the high-order mechanical resonances on the noise and distortion. However, there is no barrier to establishing the EM-ΣΔM system with other orders using the MDOF-EM model of the sensing element.

**Figure 3:**
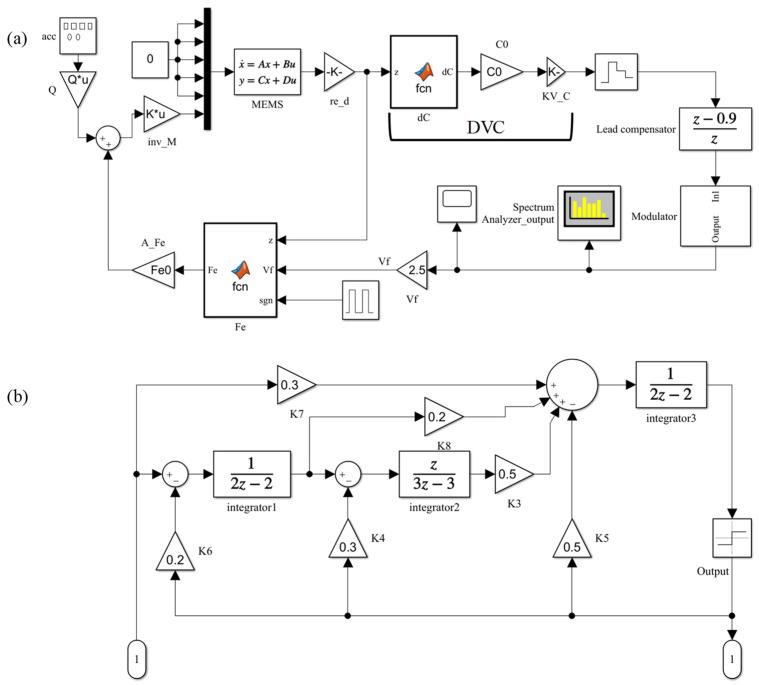
EM-ΣΔM system based on MDOF-EM model of the sensing element. (**a**) Overall system; (**b**) Subsystem of the sigma–delta modulator. DVC denotes the displacement–voltage conversion block.

The established MDOF EM-ΣΔM system is shown in Figure 3, where the fifth-order one-bit feedforward and distributed feedback (DFFF) topology diagram proposed in [32] is adopted. The system consists of a MEMS sensing element, displacement–voltage conversion, a lead compensator, a third-order modulator, and an electrostatic force block. The parameters for the lead compensator and third-order modulator were supplied by our interface circuit suppliers and are described in [32]. In future, the interface circuit will be integrated with the MEMS sensing element to implement the experimental validation. Time-multiplexing feedback technology is adopted in the EM-ΣΔM system, so a pulse generator is used to control the feedback phase.

## 4. Simulation and Discussion of Noise and Distortion

The MDOF EM-ΣΔM system is simulated, and the result is compared with that of the SDOF EM-ΣΔM system, which is established based on the SDOF-EM model of the sensing element given in Equations (29) and (31). The parameters adopted in the system are listed in Table 1. With the parameters listed in Table 1, Brownian noise can be computed using the formula from [8], and the result is 23.6 ng/√Hz.

**Figure 4:**
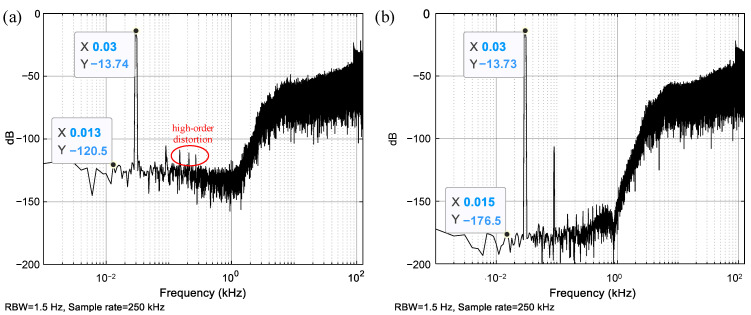
Simulated PSD. (**a**) MDOF EM-ΣΔM system; (**b**) SDOF EM-ΣΔM system.

The input acceleration has an amplitude of 0.5 g and a frequency of 30 Hz. The sampling frequency *f_s_* is 250 kHz. The simulated power spectrum density (PSD) of the one-bit output bitstream is shown in Figure 4. The low-frequency noise obtained from the MDOF system is approximately −120.5 dB, much higher than that obtained from the SDOF system, which is around −176.5 dB. The harmonic distortion obtained from the MDOF system is also more severe than that obtained from the SDOF system, especially for the high-order harmonic distortion terms. Compared to the SDOF system, the only improvement in the MDOF system is that the high-order natural modes of the sensing element are included. The lead compensator and third-order modulator of the MDOF system match those of the SDOF system. Thus, the noise and distortion degeneration must be induced by the high-order mechanical resonances of the sensing element.

**Figure 5:**
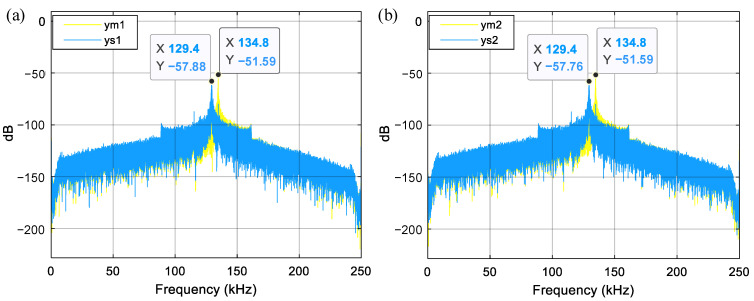
PSD of finger displacements. (**a**) Fingers in *C*_1_; (**b**) Fingers in *C*_2_.

**Figure 6:**
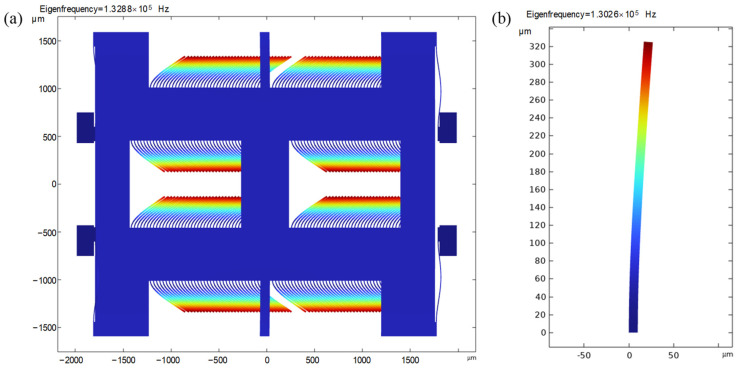
High-order natural modes of the sensing element. (**a**) High-order natural mode in proof-mass and movable fingers; (**b**) High-order natural mode in the static finger. The color closer to the red represents that the displacement is larger, while the color closer to the blue represents that the displacement is smaller.

To explain why the high-order mechanical resonances can increase the noise and distortion, the PSD of the finger displacements is studied first. The PSDs of the moveable and static finger displacements in capacitors *C*_1_ and *C*_2_ are shown in Figure 5a,b, respectively. The static finger displacement peaks at about 129.4 kHz. Another peak occurs for the movable finger displacement at about 134.8 kHz. The natural frequency simulation of the sensing element shown in Figure 6 verifies that two high-order natural modes exist around 130 kHz. The finger displacements related to the two higher-order natural modes are also massive. The peaks of the finger displacement PSDs show that the high-order mechanical resonances of the sensing element are excited in the high-vacuum environment.

Secondly, the steady-state relationship between the output and the input acceleration is derived. In a closed-loop accelerometer, the spring restoring force is minimal, so the equilibrium of proof mass is achieved using electrostatic force and inertial force:

(40) $$F_{ep}/2-ma=0$$

where the factor of (1/2) is the duty cycle of the electrostatic force feedback phase. Because the normalized displacement of proof-mass ${\tilde{y}}_{p}$ is much smaller than 1, the electrostatic force given in Equation (21) can be approximated as

(41) $$F_{ep}\approx F_{e0}{\left(S_{o}-1\right)}^{2}\left(1-1/{\tilde{D}}^{2}+a\right)-F_{e0}{\left(S_{o}+1\right)}^{2}\left(1-1/{\tilde{D}}^{2}-b\right)$$

where the coefficients *a* and *b* denote the displacement terms from fingers.

(42) $$\begin{array}{l} a=2\left(r_{m}^{T}{\tilde{y}}_{m1}-r_{s}^{T}{\tilde{y}}_{s1}\right)\\ b=2\left(r_{m}^{T}{\tilde{y}}_{m2}-r_{s}^{T}{\tilde{y}}_{s2}\right) \end{array}$$

Substituting Equation (41) into (40) leads to

(43) $$ma=F_{e0}\left(a+b\right)/2-2F_{e0}\left(1-1/{\tilde{D}}^{2}\right)S_{o}-F_{e0}\left(a-b\right)S_{o}+F_{e0}\left(a+b\right)S_{o}^{2}/2$$

As shown in Equation (43), the finger displacement term couples with the steady-state relationship between the output and the input acceleration. Notably, there is a force term resulting from the product of output and finger displacement.

(44) $$F=-F_{e0}\left(a-b\right)S_{o}$$

Because the high-order natural modes considering the bending of the fingers are excited into resonance, the finger displacement term (*a* − *b*) must have high power around the high-order natural frequencies. On the other hand, the output *S_o_* also has high power around the high-order natural frequencies because the noise shaping of EM-ΣΔM moves the power of the output noise to the high-frequency band, as shown in Figure 4a. As a result, the product of the finger displacement term (*a* − *b*) and output *S_o_* must produce low-frequency forces in the bandwidth via frequency mixing. These low-frequency forces add extra acceleration to the bandwidth, so extra low-frequency noise appears in the output. Meanwhile, the additional acceleration at the frequency where the harmonic occurs enhances the harmonic distortion term.

## 5. Suppression of Noise and Distortion

Section 5 concludes that the high-order mechanical resonances of the sensing element can increase the noise and distortion. Increasing the damping of the MEMS sensing element can suppress the noise and distortion because higher damping results in lower resonant vibrations [34]. However, this method also increases Brownian noise, which is directly proportional to the square root of the damping coefficient [14].

As shown in Equation (44), the power of the mixed force term must decrease with the reduction in the power of the bitstream output and finger displacement. Additionally, the lower output power provides more downward finger displacement because the output is fed back into the sensing element to generate the electrostatic force applied to the fingers. Thus, the noise and distortion must decrease with the decreasing output power. As shown in Figure 4a, the output power in the frequency band around 1/2 *f_s_* is high. Additionally, because the sampling principle causes the power spectrum to repeat with a frequency period of *f_s_*, it is necessary to adjust the natural frequency of high-order modes to avoid (1/2 + *n*)*f_s_*, where *n* = 1, 2, 3,…. Secondly, the vibrational amplitude is inversely proportional to the square of natural frequency [34], so the natural frequency of high-order modes should increase to the greatest extent possible. Finally, the pulse sampling voltage may also excite the high-order modes into resonance if their natural frequencies are equal to *nf_s_*, where *n* = 1, 2, 3,…. Overall, the natural frequency of high-order modes should be increased and distanced from *nf_s_*/2, where *n* = 1, 2, 3,….

**Figure 7:**
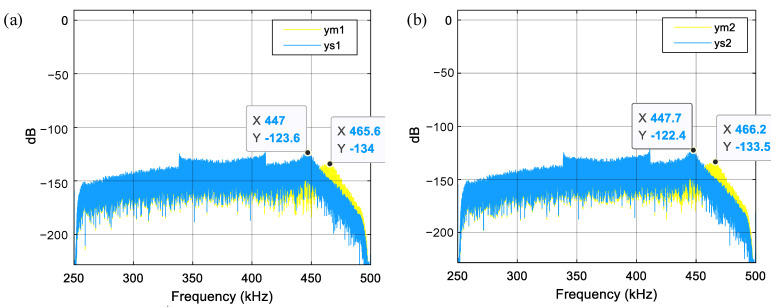
PSD of finger displacements for the system with a high-order natural frequency of about 455 kHz. (**a**) Fingers in *C*_1_; (**b**) Fingers in *C*_2_.

**Figure 8:**
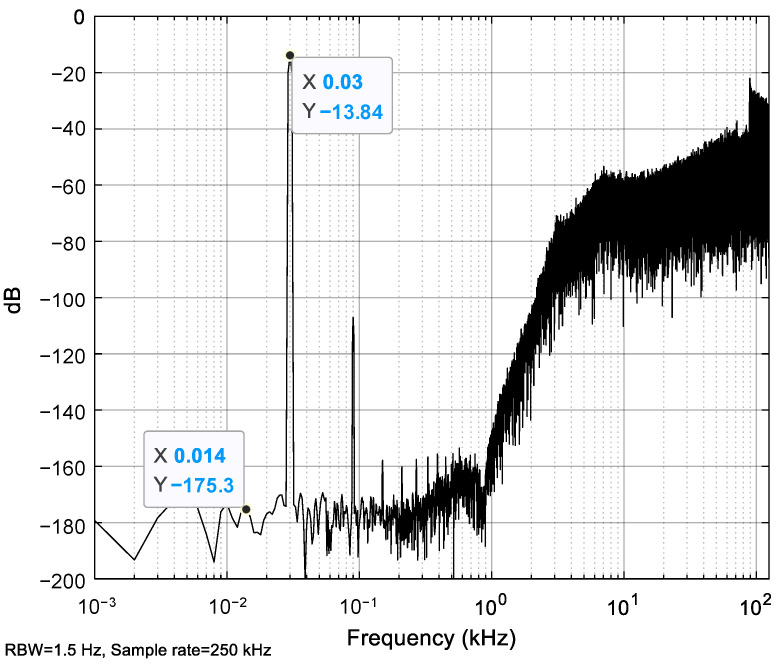
PSD of output for the system with a high-order natural frequency of about 455 kHz.

For instance, the natural frequency of high-order modes increases to about 455 kHz upon decreasing the finger length to 175 μm and increasing the number of movable fingers to 272. The natural frequency of about 455 kHz is far from *nf_s_*/2, allowing it to suppress the high-order mechanical resonances of the sensing element. With the new finger dimensions, the updated PSDs obtained from the MDOF system are shown in Figure 7 and Figure 8. Comparing the results from Figure 5 and Figure 7 reveals that the resonant peak of finger displacements decreases from about −55 dB to −125 dB after the natural frequency increases from about 130 kHz to 455 kHz. Thus, the appropriate adjustment of the high-order natural frequency effectively suppresses the high-order mechanical resonances of the sensing element. Finally, comparing the output PSDs shown in Figure 4a and Figure 8 shows that the low-frequency noise drastically decreases from about −120.5 dB to −175.3 dB, and the high-order harmonic distortion terms also decrease significantly. Notably, Figure 4b and Figure 8 show that the low-frequency noise and distortion performance have already improved to the same level as the SDOF system. Overall, carefully designing the natural frequency of the high-order modes of the sensing element is very useful for suppressing the noise and distortion induced by high-order mechanical resonances.

A comparison of this work with previously reported works is presented in Table 2. The quantization and Brownian noises obtained in this work are low. The main improvement of this work is the MDOF model that considers the fingers’ flexibility, which can be used to optimize the sensing element in future.

## 6. Conclusions

This study proposes a novel MDOF model for seismic-grade EM-ΣΔM MEMS accelerometers. The novel MDOF model was used to evaluate the noise and distortion degeneration induced by the high-order mechanical resonances of the sensing element. The simulation results and discussion from the novel MDOF model show that the high-order mechanical resonances of the sensing element produce resonant peaks of the finger displacements. Then, the product of the finger displacements and the output results in extra low-frequency forces in the bandwidth via frequency mixing. These low-frequency forces add extra acceleration to the sensing element to degrade the noise and distortion performance. Finally, to suppress the degeneration of the noise and distortion performance, the natural frequency of the sensing element’s high-order modes should be increased and distanced from *nf_s_*/2, where *n* = 1, 2, 3, …. The results show that the low-frequency noise drastically decreases from approximately −120.5 dB to −175.3 dB after the high-order natural frequency increases from about 130 kHz to 455 kHz. The harmonic distortion also reduces significantly.

In future, an optimization process of the sensing element and EM-ΣΔM system should be studied based on the novel MDOF model to ensure better performance. It is also important to investigate the effect of high-order mechanical resonances of the sensing element on the stability of the EM-ΣΔM system based on the novel MDOF model.

## Figures and Tables

**Table 1:** Parameters for modeling and simulation.

Parameters	Value	Unit
Density (*ρ*)	2330	kg/m^3^
Young’s modulus (*E*)	169	GPa
Length of spring (*L_s_*)	840	μm
Width of spring (*w_s_*)	9	μm
Proof-mass (*m_p_*)	9.11 × 10^−7^	kg
Width of movable and static fingers (*w*)	10	μm
Length of movable and static fingers (*l*)	325	μm
Height of fingers (*h*)	60	μm
Number of movable or static fingers in *C*_1_ or *C*_2_ (*N*)	144	—
Narrow gap (*d*)	3	μm
Wide gap (*D*)	10	μm
Feedback voltage (*V_f_*)	2.5	V
Dielectric constant of air (ε)	8.854 × 10^−12^	F/m
Sampling frequency (*f*_s_)	250	kHz
Capacitance–voltage conversion (*KV_C*)	6.67	V/pf
Quality factor (*Q*)	2000	—
Brownian noise	23.6	ng/√Hz

**Table 2:** Comparison of this work with previously reported works.

Authors	Principle	Model Type	Quantization Noise (dB)	Brownian Noise (ng/√Hz)	Mass for Sensing Acceleration (mg)
Chae et al. [11]	Sigma–delta	SDOF	—	700	2.8
Dong et al. [25]	Sigma–delta	SDOF	−170	850	1.2
Amini et al. [13]	Sigma–delta	SDOF	−120	1000	5
Abdolvand et al. [16]	Sigma–delta	SDOF	—	50	38
Almutairi et al. [26]	Sigma–delta	SDOF	−130	278	1.62
Chen et al. [15]	Sigma–delta	SDOF	−125	278	1.62
Xu et al. [27]	Sigma–delta	SDOF	−140	30	62
Utz et al. [7]	Analog open loop	SDOF	—	100	1.86
Zhang et al. [28]	Sigma–delta	SDOF	−120	0.693	1.11 × 10^4^
This work	Sigma–delta	MDOF	−175.3	32	1.04

## Data Availability

Data would be available upon request on a personal contact with the corresponding author at the email address: xuefengwang19@fudan.edu.cn.
